# The Role of SIRT1 in Mediating Bone Marrow Edema and Its Interface With Bone Remodeling

**DOI:** 10.1002/iid3.70505

**Published:** 2026-09-23

**Authors:** Yiming Chen, Xinyue Li, Zhongji Zheng, Zhihong Liu, Tao Xu, Yuanyi Tang

**Affiliations:** ^1^ Department of Orthopaedics (Joint and Sports Medicine) Chongqing Traditional Chinese Medicine Hospital (Nanqiaosi Campus) Chongqing China; ^2^ College of Traditional Chinese Medicine Chongqing Medical University Chongqing China; ^3^ Joint Ward 2 Traditional Chinese Medicine Hospital Tongliang Chongqing China

**Keywords:** bone marrow edema, bone marrow lesions, bone metabolism, SIRT 1, treatment

## Abstract

**Background:**

Bone marrow edema (BME), a common magnetic resonance imaging (MRI) finding characterized by increased fluid signal, is associated with pain and structural deterioration in conditions such as osteoarthritis and avascular necrosis. The pathophysiology of BME involves a complex interplay of vascular leakage, inflammation, microdamage, disrupted bone remodeling, and increased marrow adiposity. Sirtuin 1 (SIRT1), an NAD^+^‐dependent deacetylase, is a critical regulator of bone homeostasis, promoting osteoblastogenesis, inhibiting osteoclastogenesis, and suppressing bone marrow adipose tissue (BMAT) formation.

**Materials and Methods:**

This review synthesizes existing experimental, preclinical, and clinical evidence concerning SIRT1 in bone homeostasis and examines its potential relationship with mechanisms implicated in BME. Particular attention is given to endothelial integrity, inflammatory signaling, oxidative stress, bone remodeling, BMAT, and mesenchymal stem cell fate.

**Results:**

Current evidence supports a biologically plausible role for SIRT1 dysfunction in the development and persistence of BME. Reduced SIRT1 activity may contribute to vascular permeability, prolonged inflammatory signaling, oxidative stress, impaired osteoblastogenesis, enhanced osteoclastogenesis, and increased marrow adiposity. Although direct evidence linking SIRT1 specifically to BME remains limited, converging indirect evidence suggests that SIRT1 activation could mitigate several core drivers of edema. SIRT1 activators, including resveratrol and NAD^+^ precursors, therefore have potential therapeutic relevance.

**Discussion:**

SIRT1 may represent a mechanistic interface between BME, inflammation, vascular dysfunction, and disturbed bone remodeling. Combination strategies targeting SIRT1 together with vascular leakage and inflammation may offer additional therapeutic potential. However, direct lesion‐level and clinical evidence is required to establish causality, define therapeutic efficacy, and validate SIRT1 as a target for preventing or resolving BME.

## Graphical abstract

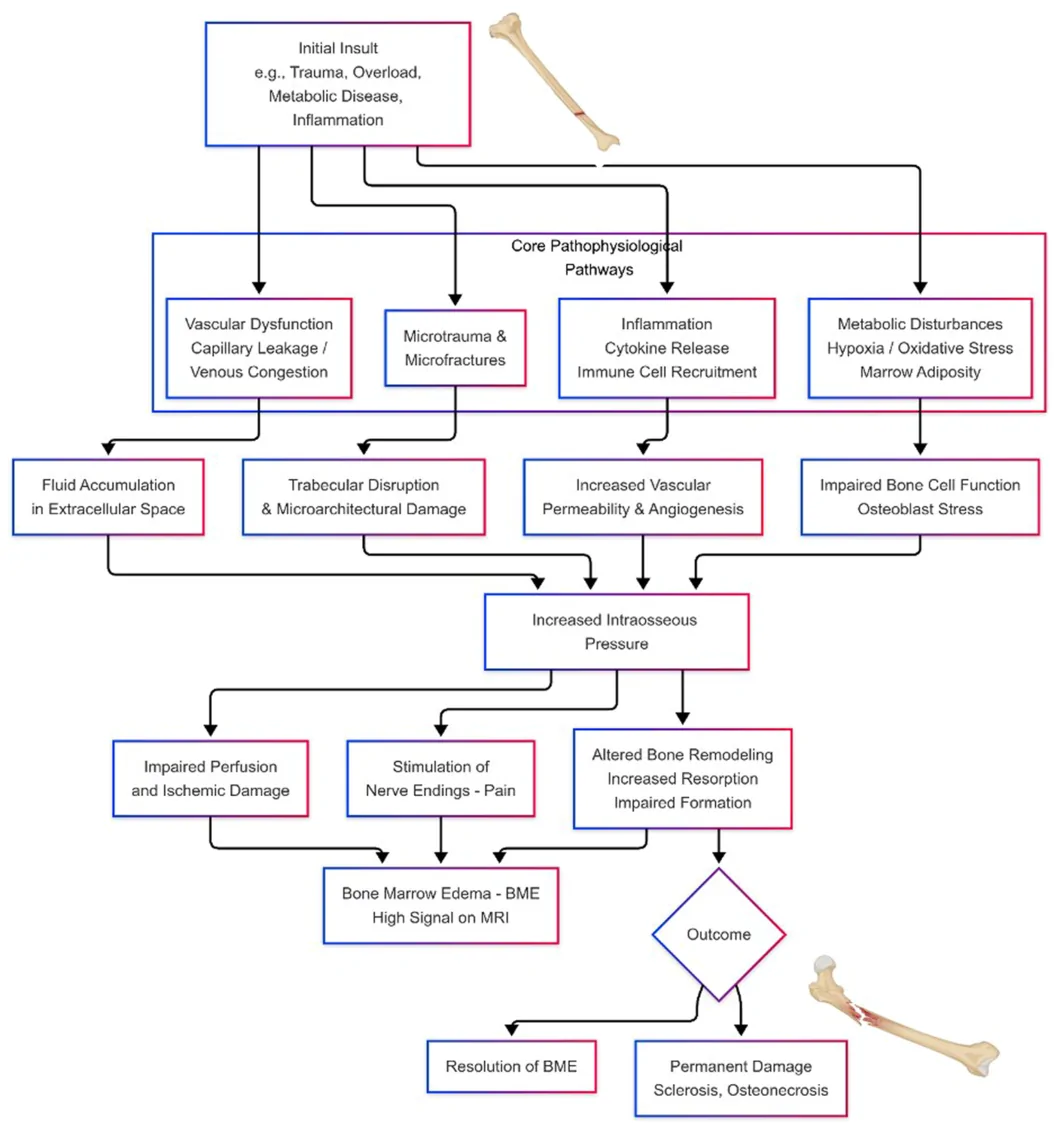

## 1 Introduction

Sirtuins are a family of conserved NAD^+^‐dependent deacetylases (and ADP‐ribosyltransferases) that regulate diverse cellular processes including metabolism, aging, inflammation, and stress responses. Among them, Sirtuin 1 (SIRT1) is one of the most well‐studied in mammals [1, 2]. It modulates chromatin structure and gene expression via deacetylation of histones and key transcription factors (such as FOXO, NF‐κB, p53), thereby influencing cell senescence, apoptosis, mitochondrial biogenesis, and metabolic homeostasis. In bone tissue, SIRT1 has been shown to support osteoblast differentiation and function, enhance progenitor cell survival, suppress osteoclastogenesis, and protect against age‐ or stress‐induced bone loss [3, 4]. Recent studies also implicate SIRT1 in regulating bone marrow adiposity via its effects on bone marrow mesenchymal stem cells (BM‐MSCs) [5, 6], dependence on NAD^+^, and modulation of metabolic gene programs (e.g. PGC‐1α, PRDM16) in marrow adipocytes. (E.g. “Sirt1 Promotes a Thermogenic Gene Program in Bone Marrow Adipocytes” demonstrates that SIRT1 activity is needed for thermogenic/adipocyte gene expression in BM‐MSCs and marrow adipose tissue [7, 8]. Similarly, in models of energy deficiency, SIRT1 deficiency leads to decreased bone mass and increased marrow adiposity, reflecting its dual role in promoting osteogenesis and suppressing adipogenesis [7, 8, 9].

Bone marrow edema (BME), sometimes interchangeably called bone marrow lesion (BML) or “edema‐like signal on MRI [10], ” refers to imaging findings characterized by increased fluid content or altered signal in the bone marrow, usually seen in MRI as hypointense signal on T1‐weighted images and hyperintense signal on T2‐weighted or STIR (short tau inversion recovery) or fat‐suppressed sequences [11]. Although MRI changes suggest increased interstitial fluid, vascular permeability, or inflammation, the exact histologic correlates of BME are heterogeneous and may include edema, fibrosis, microvascular proliferation, inflammatory infiltrates, or trabecular microdamage [12]. Clinically, BME is significant: it is associated with pain, mobility impairment, and in various diseases (e.g., osteoarthritis, rheumatoid arthritis, transient osteoporosis, joint trauma) it may predict progression, structural bone damage, or accelerated cartilage wear [13]. Causes of BME are varied and include trauma (bone contusions, microfractures), vascular compromise (ischemia, venous stasis), overload or mechanical stress, inflammatory processes, osteoarthritic changes, and sometimes unknown (primary) edema syndromes [14].

Bone remodeling is a dynamic process by which bone tissue is continuously renewed through the coordinated action of osteoblasts (which form bone), osteoclasts (which resorb bone), osteocytes (embedded cells that sense mechanical load and regulate both formation and resorption), and bone marrow stromal cells (including mesenchymal stem cells) that serve as precursors for osteoblasts and adipocytes [15, 16]. The balance of remodeling is influenced by mechanical load, hormonal signals, inflammatory mediators, metabolic state, and the local cellular niche. Bone marrow adiposity (or marrow fat) has emerged as an important component of the marrow microenvironment; increased marrow adipose tissue often correlates inversely with bone formation and is implicated in age‐ or disease‐related bone loss [17]. Disruptions in this finely tuned remodeling, whether through increased resorption, reduced formation, altered MSC differentiation, or microdamage, lead to weakened bone, osteoporosis, and an elevated risk of fracture [18].

Given these interrelated concepts, there is increasing interest in understanding whether and how SIRT1 contributes to bone marrow edema, and how marrow edema interplays with bone remodeling. SIRT1's known roles in modulating inflammation, oxidative stress, MSC differentiation, and marrow adiposity suggest plausible mechanistic links to the etiology of BME. For example, altered vascular permeability and inflammatory signaling (both of which are modulated by SIRT1) could lead to fluid accumulation in bone marrow; excessive marrow adiposity (which SIRT1 suppresses) may worsen or reflect the edematous milieu; and impaired osteoblastogenesis in SIRT1 deficiency could reduce bone formation in the wake of BME, impeding resolution. Current reviews (e.g., “Sirt1 Promotes a Thermogenic Gene Program in Bone Marrow Adipocytes” and “Sirtuin 1: a promising regulator of bone homeostasis”) summarize SIRT1's role in bone remodeling broadly, but the specific interface of SIRT1 with bone marrow edema is under‐studied [19, 20].

Thus, this review aims to collate and synthesize existing evidence regarding SIRT1 in bone biology — with special focus on edema‐linked mechanisms including inflammation, vascular changes, adiposity, and MSC fate — to propose a working model for how SIRT1 may mediate bone marrow edema and its impact on bone remodeling, and to identify key gaps for future research.

## 2 SIRT1 in Normal Bone Remodeling

SIRT1, a NAD^+^‐dependent deacetylase, is a central regulator of bone remodeling through coordinated actions on osteoblast lineage commitment and function, suppression of osteoclastogenesis, modulation of osteocyte signaling, and the integration of cellular energy metabolism with skeletal homeostasis [21, 22]. In the osteoblast lineage, SIRT1 promotes mesenchymal stem cell (MSC) proliferation and osteogenic differentiation by deacetylating and activating key transcriptional regulators. Overexpression or pharmacologic activation of SIRT1 enhances RUNX2 activity and downstream osteogenic markers such as osteocalcin, and in vivo work demonstrates that SIRT1 promotes alveolar bone formation via deacetylation and nuclear translocation of the polycomb protein Bmi1 [23], which supports MSC self‐renewal and osteoblastic commitment [7]. These molecular effects translate into increased osteoblastogenesis and bone mass in experimental models, positioning SIRT1 as a positive regulator of bone formation [24].

SIRT1 also restrains bone resorption by acting within osteoclast precursors and mature osteoclasts to inhibit NF‐κB signaling. Mechanistically, SIRT1 deacetylates the p65/RelA subunit of NF‐κB, reducing its transcriptional activity and thereby blunting RANKL‐driven osteoclast differentiation and function [21, 25]. Genetic and pharmacologic loss of SIRT1 activity increases osteoclastogenesis and bone resorption, whereas SIRT1 activation attenuates NF‐κB–dependent osteoclast programs. This repressive effect on NF‐κB is a crucial node by which SIRT1 links inflammatory and ageing‐related stimuli to altered bone resorption [26].

**Figure 1:**
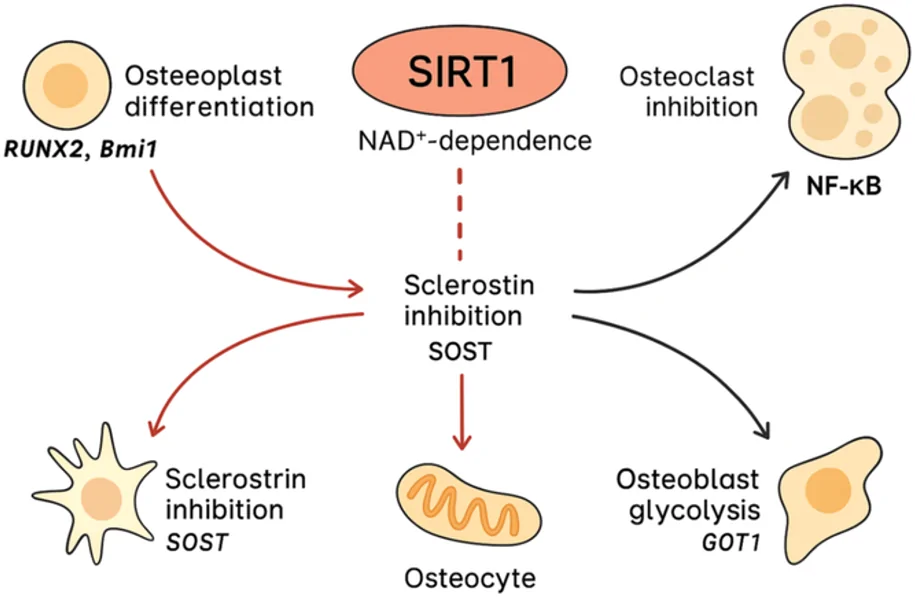
SIRT1 as a central regulator of bone remodeling. SIRT1, a NAD^+^‐dependent deacetylase, coordinates multiple aspects of skeletal homeostasis. In osteoblast lineage cells, SIRT1 promotes differentiation through activation of key transcriptional regulators (RUNX2, Bmi1). Within osteoclasts, SIRT1 suppresses NF‐κB signaling to inhibit osteoclastogenesis. In osteocytes, SIRT1 represses *Sost* transcription, reducing sclerostin secretion and thereby relieving inhibition of WNT/β‐catenin signaling in osteoblasts. SIRT1 also modulates osteoblast glycolysis (via GOT1), integrating energy metabolism with bone formation. Collectively, these actions enhance bone formation and restrain bone resorption. This figure was created using BioRender (https://BioRender.com).

Osteocytes, the principal mechanosensors of bone, contribute to SIRT1's control of bone formation through regulation of SOST (sclerostin), a potent WNT pathway antagonist [27]. Upregulation of SIRT1 in osteocytes suppresses Sost transcription, lowering sclerostin output and thereby releasing inhibition of WNT/β‐catenin signaling in osteoblasts. Molecular studies have identified a CK2–USP4–SIRT1 axis that stabilizes SIRT1 protein and represses sclerostin expression, providing a direct route by which SIRT1 activity in osteocytes modulates the balance between bone formation and resorption. Through SOST regulation, SIRT1 thus exerts an important paracrine influence on bone anabolism (Figure 1) [28].

Energy metabolism is an increasingly appreciated mechanism through which SIRT1 coordinates skeletal cell behavior with systemic metabolic state [29]. In osteoblasts, SIRT1 supports glycolytic flux and bioenergetic programs needed for matrix synthesis and mineralization. Recent work has shown that SIRT1 maintains osteoblast glycolysis via regulation of glutamic‐oxaloacetic transaminase 1 (GOT1), linking NAD^+^‐dependent deacetylation to metabolic enzyme activity and thereby preserving osteoblast function under metabolic stress [30]. Conversely, loss of SIRT1 activity shifts marrow stromal fate toward adipogenesis, increasing bone marrow adipose tissue (BMAT) and contributing to age‐related bone loss. These findings highlight SIRT1 as a metabolic rheostat in bone, integrating nutrient and redox signals with lineage decisions and anabolic capacity [31].

Together, these findings show that SIRT1 preserves bone health both via direct effects in MSCs/osteoblasts and osteoclasts, and indirectly through osteocyte‐derived factors. It promotes osteoblast differentiation (via RUNX2, Bmi1, metabolic support) while inhibiting osteoclastogenesis through NF‐κB suppression. Because SIRT1 declines with age and in metabolic disorders, reductions in its activity likely contribute to increased marrow adiposity, osteoblast senescence, elevated osteoclast activity, and heightened sclerostin production, as seen in age‐related osteoporosis. Thus, activating SIRT1 (e.g., by NAD^+^‐boosters or small molecule agonists) holds promise for restoring balance between bone formation and resorption [32], though achieving therapeutic benefit will require careful tuning of dose, timing, and cellular targeting to minimize unwanted effects.

Table 1 summarizing some key recent studies of SIRT1 in bone remodeling/osteoblast/osteoclast function. It includes the model used, the intervention or genetic modification, main findings, signaling pathways, and implications.

**Table 1:** Summary of recent studies investigating the role of SIRT1 in bone remodeling, osteoblast, and osteoclast function.

Model/system	SIRT1 manipulation/treatment	Major findings	Key pathways/markers affected	Implications for bone remodeling	References
Primary osteoblasts ex vivo (mouse) + resveratrol in vivo	Genetic deletion of **Sirt1** in osteoblasts; activation via resveratrol	Deletion reduced expression of RUNX2 downstream targets (Osx, osteocalcin, etc.), impaired osteoblast differentiation; resveratrol increased RUNX2 target expression in calvaria	RUNX2 downstream targets; transcriptional activation by SIRT1; osteoblast differentiation markers	Confirms SIRT1 as regulator of RUNX2‐mediated osteoblast differentiation; suggests activators can boost bone formation	[33]
Ovariectomized (OVX) mice + ex vivo osteoblasts under oxidative stress	Treatment with resveratrol	Resveratrol ameliorated bone loss, increased proliferation/differentiation of osteoblasts, reduced apoptosis, lowered ROS; in osteoblasts under H_2_O_2_ stress: resveratrol protected cells	Increased SIRT1, increased nuclear FoxO1; increased antioxidant enzyme (SOD); lower ROS; osteoblast markers elevated	Highlights that SIRT1 activation counters oxidative stress and improves osteoblast survival and function in osteoporotic conditions	[34]
Whole‐body, osteoblast‐specific, and osteoclast‐specific **SirT1** knockout mice; also calorie restriction model	Knockout of Sirt1 in different lineages; resveratrol/calorie restriction as physiologic activator	SirT1 KO mice: low bone mass, fewer osteoblasts, increased osteoclasts; osteoblast‐specific KO reduced osteoblasts; osteoclast‐specific KO increased resorption; caloric restriction improved bone mass in WT but not Sirt1 KO	NF‐κB (especially p65/RelA) repression in osteoclasts; RUNX2 transactivation in osteoblasts; linkage to metabolic signals (CR)	Demonstrates dual role of SIRT1: both promoting bone formation and inhibiting resorption; supports metabolic modulation of bone via SIRT1	[35]
Osteoblast‐specific Sirt1 conditional knockout (cKO) mice; also in vitro osteoblasts	Deletion of Sirt1 in osteoblasts (inducible)	cKO mice had lower trabecular and cortical bone mass; decreased bone formation and resorption; in vitro, osteoblast differentiation, mineralization reduced; apoptosis increased	Metabolomics + acetylome: decreased glycolysis; key substrate GOT1 identified; increased acetylation of glycolysis‐related proteins; osteoblast markers (Runx2, Col1a1, Alpl, Bglap) reduced	Suggests that SIRT1 supports energy metabolism in osteoblasts (via glycolysis), which is critical for their function; metabolic impairment may underlie bone deficiency when SIRT1 is low	[36]
Human MSCs/preosteoblastic cells in vitro	Treatment with osteogenic induction + resveratrol; inhibition of SIRT1 by nicotinamide; knockdown experiments	Resveratrol enhanced expression of Runx2 and osteogenic markers; inhibited PPAR‐γ (adipogenic) expression; knockdown of SIRT1 removed these effects	RUNX2 deacetylation; PPAR‐γ suppressed; NCoR interaction; measurement of osteogenic vs adipogenic markers	Supports that SIRT1 governs MSC fate, suppressing adipogenesis, promoting osteogenesis—a relevant mechanism when marrow adiposity is increased	[37]
Bone marrow‐derived macrophages (BMMs), in vitro	Overexpression of SIRT1 or treatment with resveratrol; exposure to TNF‐α	SIRT1 downregulation seen during osteoclastogenesis; SIRT1 overexpression/activation inhibited formation of multinucleated osteoclasts, reduced TRAP activity; lowered expression of osteoclast genes	ROS generation; TRPV1 activation; osteoclast marker genes down; NF‐κB possibly implicated	Adds evidence that SIRT1 suppresses osteoclastogenesis under inflammatory stimuli; could be protective in conditions with elevated TNF‐α	[38]

## 3 Mechanisms Underlying Bone Marrow Edema (BME)

Bone marrow edema (BME), often visualized as high signal on fluid‐sensitive MRI sequences, reflects a spectrum of histopathologic and physiologic changes rather than a single uniform process. Recent work has clarified several overlapping pathophysiological mechanisms that contribute to its development: vascular leakage and increased intraosseous pressure, micro‐fractures, inflammation, and disruption of normal bone homeostasis including turnover, hypoxia, oxidative stress, and marrow adiposity [39].

### 3.1 Pathophysiology of BME

One central feature is vascular dysfunction. Capillary leakage in bone marrow causes fluid to escape into extracellular marrow spaces; this leads to interstitial fluid accumulation. In non‐traumatic or degenerative settings, impaired drainage of intraosseous capillaries or venous congestion can further exacerbate fluid buildup [40].

Increased intraosseous pressure follows from fluid accumulation and sometimes hemorrhage. As pressure rises, this can irritate nerve endings and impair perfusion, contributing to pain and possibly ischemic damage. Microtrauma and microfractures—small disruptions in trabecular bone (or subchondral bone plates) are also frequently implicated, either as the initiating event (in trauma, overuse) or as secondary damage via mechanical overload [41].

Inflammation is another major component. Local inflammatory responses release cytokines, increase vascular permeability, recruit immune/inflammatory cells, and promote angiogenesis. This inflammation both contributes to fluid accumulation and may provoke changes in bone remodeling (resorption and formation) depending on context [42].

### 3.2 Relationship Between BME and Bone Remodeling

BME is not just a passive imaging finding: it often correlates with altered markers of bone turnover. For example, in osteoarthritis and subchondral bone marrow lesion (BML) contexts, sites of edema show increased bone formation and resorption activity when compared to adjacent unaffected areas. Such turnover is evident both in imaging (bone thickening, sclerosis, changes in trabecular microarchitecture) and in biochemical markers. When remodeling is sufficient, some lesions resolve; if not, more permanent changes like sclerosis, microarchitectural deterioration and even osteonecrosis can follow [43].

Microarchitectural changes include increased trabecular spacing, thinning of trabeculae, and loss of connectivity in the cancellous bone, particularly in subchondral regions. In knee OA for example, these changes occur early, often in conjunction with edema signals, before cartilage degradation becomes advanced [43].

### 3.3 Role of Marrow Adiposity, Hypoxia, and Oxidative Stress

Marrow adiposity (BMAT) is emerging as an intermediary in the BME‐remodeling link. Increased marrow fat in aging, metabolic disease, or disuse can impair local vascular supply, reduce effective oxygenation, and alter secretion of adipokines which may have proinflammatory or anti‐osteogenic effects. Studies show that areas of high BMAT often have lower bone formation or higher resorption markers, and may be more prone to edema or lesions under stress [44].

Hypoxia also contributes: when perfusion is compromised (due to vascular leakage, capillary collapse, or increased pressure), oxygen delivery falls, which leads to hypoxic stress. Hypoxia induces expression of factors such as VEGF to promote angiogenesis, but also can stimulate osteoclast activity and impair osteoblast function. In addition, oxidative stress driven by reactive oxygen species (ROS) generated under hypoxic conditions, metabolic dysregulation, or inflammation damages bone cells, triggers cell death or senescence, and disrupts the balance of remodeling [45]. A recent study in avascular necrosis of the femoral head (ONFH) observed that pre‐collapse BME is associated with increased oxidative stress, fibrosis, and elevated osteoclast activity [46].

**Figure 2:**
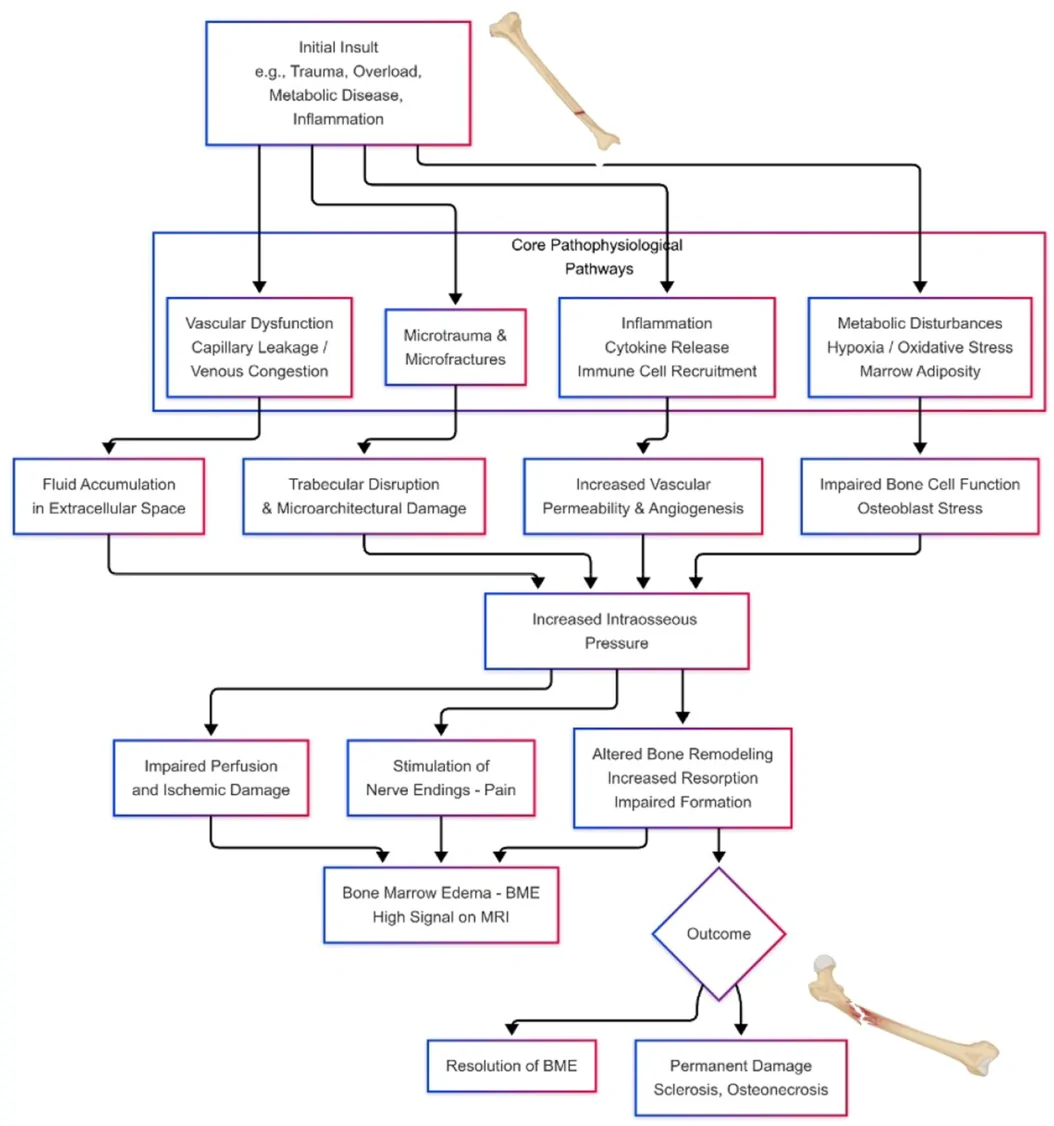
Pathophysiological mechanisms of Bone Marrow Edema (BME). An initial insult (e.g., trauma, metabolic disease) triggers four core, interlinked pathways: 1) Vascular dysfunction leading to fluid accumulation; 2) Microtrauma causing structural damage; 3) Inflammation increasing permeability; and 4) Metabolic disturbances (hypoxia, oxidative stress, marrow adiposity) impairing bone cell function. These processes converge to increase intraosseous pressure, which causes pain, impairs perfusion, and disrupts bone remodeling. The imbalance between bone resorption and formation dictates the outcome, leading either to the resolution of BME or progression to permanent structural damage.

Bone Marrow Edema is a multifactorial phenomenon arising from vascular leakage, microdamage, inflammation, and the confluence of metabolic factors like hypoxia, oxidative stress, and marrow adiposity. These processes interweave to disrupt the equilibrium of bone remodeling: resorption increases, formation lags, microarchitecture deteriorates, and clinical symptoms such as pain ensue. Understanding these mechanisms is crucial because some BME lesions resolve with interventions (rest, loading modification, anti‐inflammatories), while others progress to irreversible structural damage like osteonecrosis or permanent bone loss. Figure 2 shows the pathophysiological mechanisms of BME.

## 4 Evidence Linking SIRT1 to BME or Edema‐Like Conditions

Direct evidence that SIRT1 expression or enzymatic activity is altered specifically in bone marrow edema (BME) lesions in humans or animals is limited. To date, few studies have measured SIRT1 levels within BME/BML (bone marrow lesion) tissue sampled from patients or used imaging‐guided biopsies to correlate local SIRT1 activity with edema signals on MRI. Several recent reviews and primary reports therefore highlight an evidence gap: SIRT1 is implicated broadly in bone homeostasis, but direct, lesion‐level data tying SIRT1 dysregulation to BME are scarce and represent an important unmet need for the field [47].

Because direct data are limited, most evidence linking SIRT1 to edema‐like bone states is indirect and builds from well‐established SIRT1 roles in cell types and processes known to participate in BME pathogenesis [48]. First, SIRT1 strongly influences bone marrow mesenchymal stem cell (BM‐MSC) fate decisions: it promotes osteogenic differentiation and suppresses adipogenic programs via deacetylation of β‐catenin and other transcriptional regulators, shifting the BM‐MSC lineage toward bone formation rather than marrow adiposity. These cell‐autonomous effects imply that reduced SIRT1 activity could favor marrow adipogenesis and an environment prone to edema and altered remodeling [49].

Second, bone marrow adiposity (BMAT) itself is mechanistically linked to edema and poor bone remodeling. Experimental and translational studies show that increased BMAT correlates with impaired perfusion, altered cytokine/adipokine secretion [50], and reduced osteoblast activity all of which can predispose marrow compartments to fluid accumulation and microenvironmental dysregulation [51]. Multiple mouse models demonstrate that SIRT1 deficiency or downregulation increases BMAT while reducing bone mass, suggesting a pathway by which loss of SIRT1 could promote edema‐prone marrow states. One relevant animal model a separation‐based chronic energy‐deficit (anorexia‐like) model produced increased BMAT and bone loss in Sirt1‐deficient mice, and resveratrol (a SIRT1 activator) partially rescued these phenotypes, linking energy deficit, reduced SIRT1, marrow adiposity, and poor bone architecture [52].

Third, SIRT1 modulates inflammation, oxidative stress, and vascular responses central mechanisms in BME pathophysiology. SIRT1 deacetylates NF‐κB components and attenuates proinflammatory cytokine production in multiple tissues; it also enhances cellular defenses against oxidative stress and can influence angiogenic signaling. Given that BME commonly follows microvascular leakage, inflammatory cell infiltration, and ROS‐mediated tissue injury, diminished SIRT1 activity could exacerbate vascular permeability, prolong inflammatory signaling, and impair redox homeostasis within marrow, thereby promoting or sustaining edema [21].

While energy‐deficit or anorexia‐like models (e.g., separation‐based anorexia models) provide valuable insights into the relationship between SIRT1, bone marrow adiposity (BMAT), and bone remodeling [47, 53], it is important to recognize their limitations in the context of bone marrow edema (BME). These models predominantly reflect chronic metabolic stress and increased marrow adiposity [53], but may not adequately recapitulate key features of BME observed in clinical settings, such as acute vascular leakage, inflammatory cell infiltration, or trauma‐induced microdamage [54]. As such, although they support a role for SIRT1 in shaping marrow composition and metabolic environment, their relevance to the acute and heterogeneous pathophysiology of BME remains indirect. Future studies employing models that more closely mimic vascular dysfunction and inflammatory components of BME will be essential to establish translational relevance.

Therapeutic modulation of SIRT1 provides complementary, translationally relevant evidence. Small‐molecule SIRT1 activators (resveratrol and related compounds) have been shown in numerous preclinical bone models to enhance osteoblast function, reduce BM‐MSC adipogenic differentiation, and improve bone mass or microarchitecture. In energy‐deficit and osteoporotic models, resveratrol restored markers of bone formation, reduced marrow fat, and ameliorated structural bone deterioration in a SIRT1‐dependent manner. While these studies do not measure edema per se, they show that manipulating SIRT1 shifts the marrow milieu away from adiposity, inflammation, and bone fragility conditions that, when present, commonly associate with BME on imaging [55].

Despite the convergent indirect evidence, caution is warranted. First, causality between altered SIRT1 and BME has not been proven: changes in SIRT1 may be a downstream response to metabolic stress, hypoxia, or inflammation rather than the initiating driver of edema. Second, heterogeneity among BME etiologies (traumatic, osteoarthritic, ischemic/AVN, inflammatory) means SIRT1's relevance may vary by context. Finally, human clinical data linking SIRT1 activity (measured in marrow biopsies, serum markers, or imaging‐biomarker correlations) to BME severity, duration, or prognosis are essentially absent — an important gap for translational validation [25].

**Figure 3:**
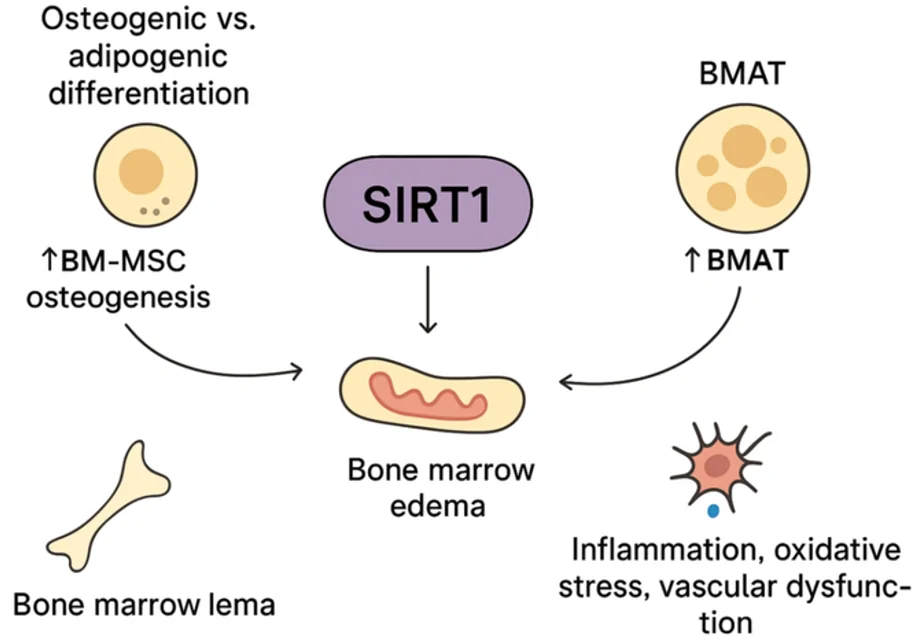
Proposed molecular links between reduced SIRT1 activity and bone marrow edema (BME). Lower SIRT1 expression or enzymatic activity in bone marrow mesenchymal stem cells (BM‐MSCs) may shift lineage commitment away from osteogenesis toward adipogenesis, resulting in increased bone marrow adipose tissue (BMAT), impaired osteoblast function, and an edema‐prone marrow environment. In parallel, diminished SIRT1 activity can exacerbate inflammation, oxidative stress, and vascular dysfunction—factors that further contribute to marrow fluid accumulation and bone remodeling defects. While direct lesion‐level evidence is lacking, these pathways suggest a biologically plausible connection between SIRT1 dysregulation and BME pathophysiology. This figure was created using BioRender (https://BioRender.com).

Current evidence supports a biologically plausible connection between reduced SIRT1 signaling and marrow states that favor edema, principally via increased BMAT, impaired osteogenesis, heightened inflammation, oxidative stress, and disturbed vascular function. However, direct lesion‐level data in BME are lacking. Future work should (1) measure SIRT1 expression/activity in human and animal BME lesions, (2) use conditional Sirt1 modulation to test causality for edema formation and resolution, and (3) evaluate whether SIRT1 activators can prevent or resolve BME in clinically relevant models. These studies would clarify whether SIRT1 is a viable therapeutic target for edema‐associated bone disease. Figure 3. Illustrate the proposed molecular links between reduced SIRT1 activity and BME.

## 5 Potential Molecular Pathways by Which SIRT1 Could Influence BME

SIRT1 (Silent Information Regulator Type 1) is implicated in several molecular pathways that could influence the development or resolution of bone marrow edema (BME) [56]. Although direct studies in BME are scarce, multiple lines of evidence from endothelial biology, MSC fate, inflammation, oxidative stress, and metabolic regulation suggest plausible mechanistic links. Below I outline four key molecular routes through which SIRT1 may modulate components of edema within bone marrow.

### 5.1 Regulation of Vascular/Endothelial Integrity in Marrow

Endothelial cell function and capillary integrity are central to preventing vascular leakage, one of the hallmarks of BME. SIRT1 promotes endothelial health via regulation of eNOS (endothelial nitric oxide synthase), antioxidant defenses, and maintenance of the endothelial glycocalyx.

SIRT1 deacetylates eNOS, enhancing its activity, thereby increasing nitric oxide (NO) production, which maintains vasodilation, prevents excessive shear stress, and reduces vascular permeability. Dysfunctions in eNOS are associated with leaky vasculature [57]. The endothelial glycocalyx, a layer of proteoglycans and glycoproteins lining blood vessels, helps to maintain barrier function. When SIRT1 activity is reduced in endothelial cells, the glycocalyx is degraded; loss of SIRT1 deacetylase activity has been shown in mice to reduce glycocalyx volume, increase superoxide production and elevate acetylated NF‐κB/p65, which likely contribute to increased permeability and capillary leakage [58]. SIRT1 also supports vascular integrity through regulation of angiogenesis and endothelial survival. For example, activation of SIRT1 helps prevent apoptosis in marrow endothelial cells under oxidative stress and supports mitochondrial function. A recent study in bone marrow endothelial cells (BMECs) demonstrated that erythropoietin treatment induced nuclear translocation of SIRT1, maintaining endothelial integrity via modulation of mitochondrial health [59].

These vascular mechanisms suggest that loss of SIRT1 could exacerbate capillary leakage, reduce barrier function, and thus contribute to fluid accumulation characteristic of BME.

### 5.2 Inflammatory Signaling, Oxidative Stress, ROS, HIF‐α Pathways

Inflammation and oxidative stress are strongly implicated in BME. SIRT1 is known to suppress inflammatory signaling and ROS production, and also interacts with hypoxia‐induced pathways.

**Figure 4:**
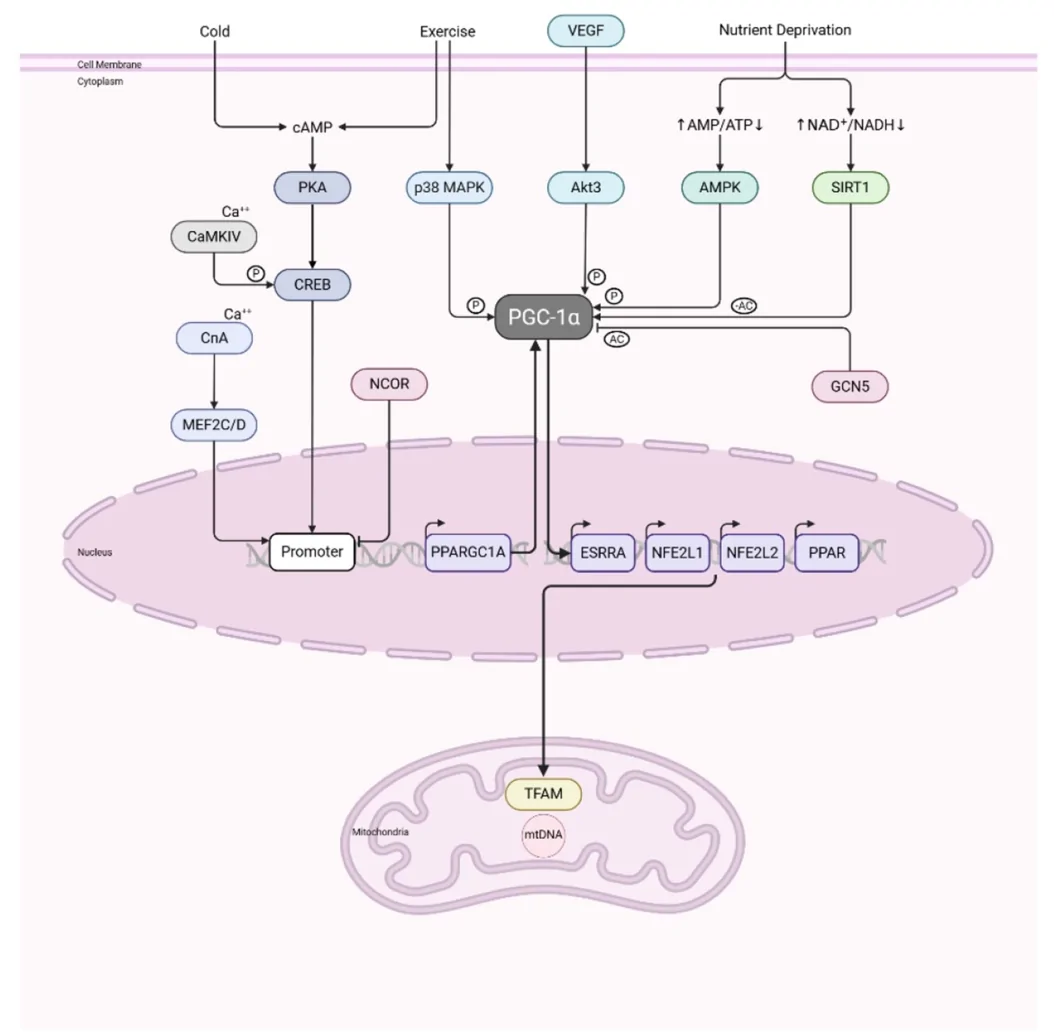
SIRT1 regulates inflammation and oxidative stress in the bone marrow by deacetylating NF‐κB p65/RelA to suppress proinflammatory cytokines, enhancing Nrf2 and PGC‐1α activity to boost antioxidant responses, and potentially influencing HIF‐1α pathways under hypoxic conditions. This figure was created using BioRender (https://BioRender.com).

SIRT1 deacetylates NF‐κB components (e.g., p65/RelA) and suppresses transcription of proinflammatory cytokines such as IL‐1β, IL‐6, and TNF‐α [56]. In bone marrow MSCs, TNF‐α induces inflammatory mediators; miR‐128‐3p has been shown to reduce SIRT1 expression in BM‐MSCs, thereby increasing IL‐6, IL‐1β, MMP9, MCP‐1 etc., while resveratrol (a SIRT1 activator) can ameliorate these changes [60]. Oxidative stress is also controlled via SIRT1 modulation of antioxidant response pathways: SIRT1 deacetylates and enhances activity of Nrf2 (a master regulator of antioxidant genes), PGC‐1α, FOXO family transcription factors, contributing to elevated expression of enzymes like SOD, catalase, HO‐1. In BMECs, SIRT1 helps preserve mitochondrial function under oxidative stress [61]. Hypoxia‐inducible factor (HIF‐α) pathways are relevant in low‐oxygen marrow regions or when perfusion is compromised due to vascular leakage or high intraosseous pressure. Although studies connecting SIRT1 directly to HIF in marrow edema are limited, SIRT1 is known elsewhere to regulate HIF‐1α stability, and may thereby modulate responses to hypoxia, including vascular endothelial growth factor (VEGF) expression, angiogenesis, and possibly capillary permeability (Figure 4) [62].

### 5.3 MSC Differentiation Balance: Osteogenesis vs Adipogenesis

**Figure 5:**
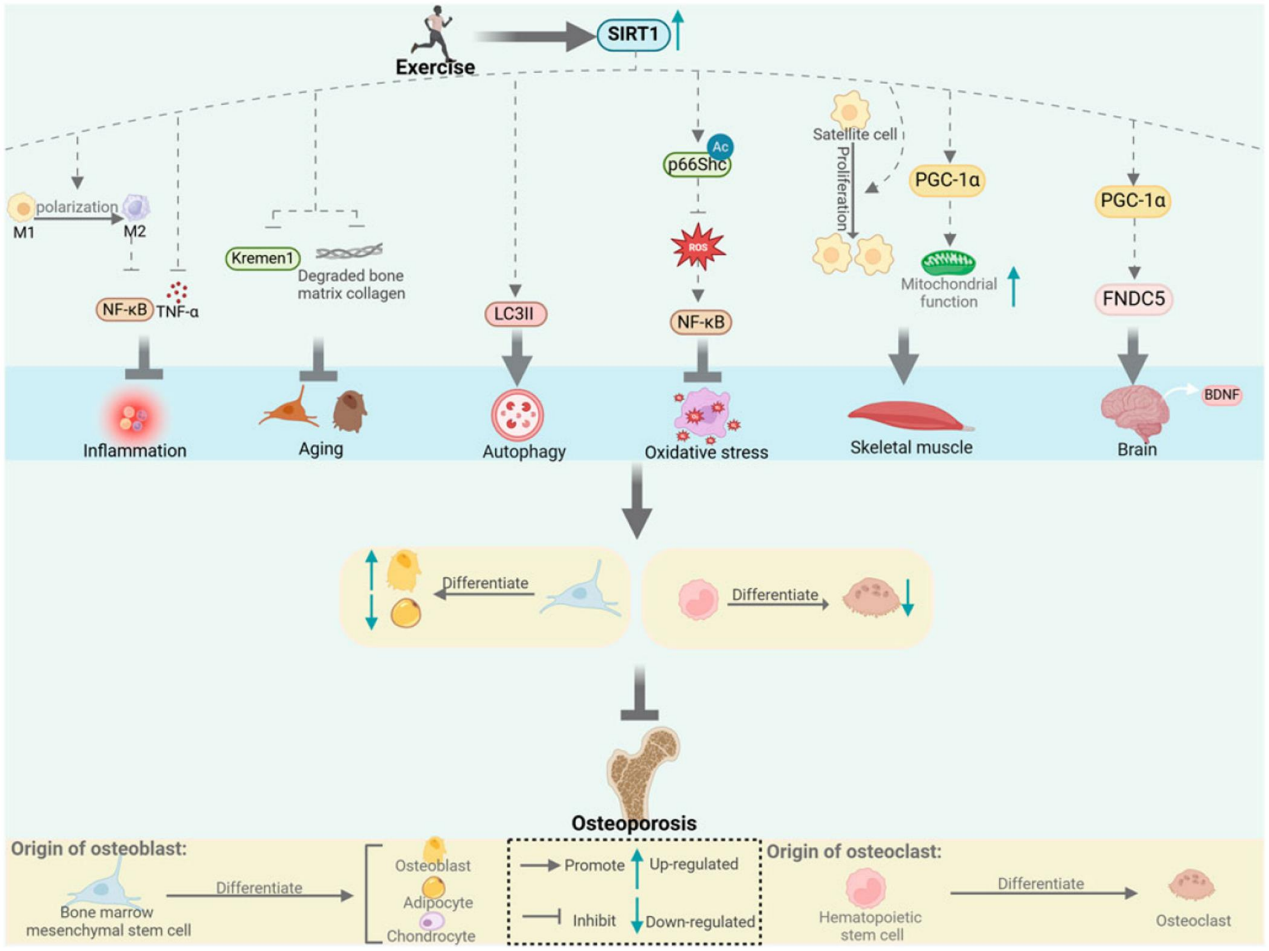
Exercise influences bone metabolism through the activity of SIRT1, a protein that regulates various cellular processes. SIRT1 modulates bone metabolism by affecting inflammation, aging, autophagy, oxidative stress, and through interactions between muscle and bone, as well as nerve and bone. Reproduced with permission from [63].

The balance between osteogenesis (bone formation) and adipogenesis (fat formation) in the marrow influences structural integrity, marrow volume, and possibly space for fluid accumulation. SIRT1 promotes osteogenic differentiation and suppresses adipogenic differentiation of BM‐MSC. Reduction in SIRT1 shifts the balance toward adipocyte expansion. Enlarged BMAT can compress vascular spaces, impair blood flow, and secrete adipokines that promote inflammation or vascular dysfunction factors that could exacerbate edema [63]. Exercise‐induced stimuli studies show upregulation of SIRT1 in BM‐MSCs and osteoblasts, which is associated with reduced oxidative stress, less adipogenesis, improved mitochondrial function, and improved bone microarchitecture (Figure 5) [63]. These changes plausibly reduce risk or severity of edema.

### 5.4 Effects on Metabolic Support: Glycolysis, Mitochondrial Function, Fluid Homeostasis

Energetic metabolism in stromal and bone cells is a less‐explored but potentially critical dimension in BME pathophysiology.

SIRT1 enhances mitochondrial biogenesis via deacetylation of PGC‐1α, regulation of oxidative phosphorylation, and supports mitochondrial health under stress. Marrow endothelial and stromal cells with healthier mitochondria are better able to maintain ion gradients, respond to hypoxia, and clear reactive species [64]. Although specific recent studies linking SIRT1 to glycolysis in BMECs or MSCs in BME are limited, SIRT1 is known to foster glycolytic metabolism in other cell types during conditions of stress, enabling energy production when oxygen is limiting or mitochondrial dysfunction occurs [65]. Efficient glycolysis and mitochondrial function could mitigate edema by maintaining cell volume regulation, reducing accumulation of metabolic byproducts that draw fluid, and supporting ion pumps (e.g., Na^+^/K^+^‐ATPase) that control fluid movement [66].

SIRT1 also interacts with nutrient‐sensing pathways like AMPK and mTOR; activation of SIRT1 often goes along with AMPK activation, suppression of mTORC1, promotion of autophagy. These help cells survive metabolic stress, clear damaged organelles, reduce ROS, maintain barrier function [67].

In summary, based on recent studies, SIRT1 has multiple molecular pathways through which it could influence BME or edema‐like states in bone marrow. These include preserving endothelial integrity (via eNOS, glycocalyx, mitochondrial protection), restraining inflammatory and ROS pathways, shifting MSC fate away from adipogenesis and toward osteogenesis, and maintaining metabolic resilience of marrow stromal/endothelial cells. Direct experimental confirmation in BME lesions remains sparse, but the convergence of these mechanisms makes SIRT1 a promising candidate for further investigation in the pathogenesis and resolution of BME.

### 5.5 Imaging Modalities in Bone Marrow Edema

Bone marrow edema (BME) is most commonly detected using magnetic resonance imaging (MRI) [40], where it appears as areas of increased signal intensity on fluid‐sensitive sequences and serves as a sensitive marker of underlying bone injury, inflammation, or vascular dysfunction. However, recent advances in imaging technology have highlighted the potential of spectral computed tomography (CT) as an alternative modality for visualizing BME [68].

Spectral CT, particularly through techniques such as virtual non‐calcium (VNCa) imaging and three‐material decomposition, enables the differentiation of marrow components and allows indirect visualization of increased water content within bone [69]. In a recent study by Schierenbeck et al. 2023 Diagnostics spectral CT BME study [70], spectral CT demonstrated high diagnostic performance for detecting BME, with sensitivities ranging from approximately 86%–94% and specificities from 84% to 94%, approaching the accuracy of MRI in certain anatomical regions.

Importantly, spectral CT offers practical advantages in acute clinical settings, including rapid acquisition, wider availability, and fewer contraindications compared to MRI [71]. This makes it particularly valuable in trauma or emergency scenarios where timely diagnosis is critical. Furthermore, advances in quantitative imaging of marrow composition may facilitate future studies linking molecular regulators, such as SIRT1, with spatially resolved BME lesions. Integration of advanced imaging modalities with molecular and metabolic profiling could therefore enhance understanding of BME pathophysiology and support the development of targeted therapeutic strategies.

## 6 Therapeutic Implications

### 6.1 SIRT1 Activators, Small Molecules, and NAD^+^ Precursors

One of the most obvious therapeutic strategies is activation of SIRT1 via small molecules like resveratrol or synthetic activators, or boosting intracellular NAD^+^ levels (since SIRT1 is NAD^+^‐dependent). Resveratrol has been studied in multiple animal models of bone loss or metabolic disease: in ovariectomized rodents, resveratrol improves trabecular bone structure, reduces osteoclast activity, and enhances osteoblast differentiation via mechanisms such as the SIRT1/β‐catenin pathway [25]. In human T2DM (Type 2 diabetes mellitus) patients, resveratrol supplementation increases SIRT1 expression in BM‐MSCs, reduces apoptosis, and promotes osteogenic differentiation [72]. Although none of these studies directly test BME, the changes are relevant: decreased marrow adiposity, improved bone microarchitecture, reduced inflammation, and enhanced metabolic competence could all reduce factors that promote edema [72].

NAD^+^ precursors (e.g., nicotinamide mononucleotide, nicotinamide riboside) or other SIRT1‐activating compounds are less well studied in bone marrow or in contexts of edema. However, enhancing NAD^+^ availability has shown in other tissues to improve endothelial function, reduce oxidative stress, and support mitochondrial health all of which are plausible routes to prevent vascular leakage and inflammation that might drive BME [73].

### 6.2 Combining Therapies: Targeting Vascular Leakage and Inflammation

Given the multifactorial nature of edema, combining SIRT1 activation with agents that directly address vascular permeability or inflammatory cytokines could have additive or synergistic effects. For example, therapies aimed at restoring endothelial barrier (via eNOS, tight junction proteins) or inhibitors of VEGF overexpression might mitigate vascular leakage [74]. Parallel control of IL‐1β, TNF‐α, IL‐6 signaling (via antibodies, small molecule inhibitors) together with SIRT1 activators may reduce inflammatory stimulus that drives fluid accumulation. Though no published studies (to our knowledge) have tested such combination therapies specifically for BME, in analogous models (e.g., osteoarthritis, metabolic bone disease), combinations of anti‐inflammatory agents plus resveratrol‐like compounds produce stronger protection than either alone [25]. Importantly, the potential for adverse skeletal effects at higher doses highlights the need for cautious optimization of SIRT1‐targeting therapies, particularly in the context of BME where both edema resolution and structural integrity must be preserved.

A major translational challenge in targeting SIRT1 is achieving therapeutically effective concentrations within the highly specialized bone marrow microenvironment [32]. The bone marrow niche, particularly its capillary and sinusoidal network, presents unique barriers to drug delivery, including limited perfusion gradients, rapid systemic clearance, and restricted penetration into trabecular regions [75]. These limitations are especially relevant for natural SIRT1 activators such as resveratrol, which exhibit poor bioavailability and rapid metabolism.

Emerging strategies such as nanoparticle‐based delivery systems, liposomal formulations, and bone‐targeting conjugates may offer potential solutions by improving pharmacokinetics, enhancing tissue‐specific accumulation, and enabling controlled release within the marrow microenvironment [76]. However, these approaches remain largely experimental in the context of skeletal disorders, and their efficacy in targeting SIRT1 within bone marrow edema (BME) lesions has yet to be established. Further work is required to bridge this gap between molecular promise and clinical feasibility.

### 6.3 Possible Risks and Side Effects

While the potential is promising, there are important risks and challenges to consider: High doses of SIRT1 activators may trigger unwanted effects: resveratrol, for instance, in some animal ageing models has been shown to reduce trabecular bone volume when used chronically at certain doses, or increase bone resorption markers in ways that may not favor net bone health [77]. Also, in chemotherapy‐induced bone injury models, resveratrol at lower doses helped, but higher doses worsened some adiposity or osteoclastogenic gene expression [77]. While therapeutic activation of SIRT1 shows promise, it is important to recognize that its effects may be dose‐dependent and context‐specific. Notably, high doses of SIRT1 activators such as resveratrol have, in some experimental settings, been associated with reduced trabecular bone volume and increased markers of bone resorption [77], which could be counterproductive in conditions characterized by structural bone fragility. This raises important considerations for BME, where preservation of bone microarchitecture is critical for lesion resolution and prevention of progression to irreversible damage.

Systemic activation of SIRT1 affects many organs; effects in marrow/endothelium need to be balanced against potential unwanted actions in other tissues (e.g., undesired metabolic or hormonal modulation, interaction with other signaling pathways). Bioavailability, pharmacokinetics: Resveratrol has relatively poor bioavailability [78]; reaching effective concentrations in bone marrow or in capillary microenvironment might require novel delivery strategies (nanoformulations, targeted delivery) [79]. The effective window for prevention or resolution of BME (acute vs chronic) may differ; early intervention might prevent edema, whereas later activation might be less effective or even counterproductive if remodeling is already disrupted. In human trials using resveratrol, most adverse events are mild (gastrointestinal discomfort, etc.) [80]. But risks in special populations (e.g. with coagulopathies, those on anticoagulants, or with other comorbid conditions) need careful assessment.

It should also be noted that much of the mechanistic evidence is derived from metabolic or energy‐deficit models, which may not fully represent the vascular and inflammatory complexity of BME in clinical contexts The excessive or non‐physiological activation of SIRT1 may disrupt the balance between bone formation and resorption, potentially exacerbating structural deterioration rather than promoting repair. Therefore, therapeutic strategies targeting SIRT1 must carefully consider dose optimization, timing of intervention, and tissue‐specific effects. Further studies are needed to define the therapeutic window within which SIRT1 activation provides benefit without inducing adverse skeletal outcomes. Table 2 highlights the therapeutic Strategies Involving SIRT1 Activation for Bone Marrow Edema and Bone Health.

**Table 2:** Therapeutic strategies involving SIRT1 activation for bone marrow edema and bone health.

Therapeutic strategy	Key compounds/agents	Proposed mechanism of action	Relevant findings and context	Key considerations/challenges
SIRT1 activators & NAD^+^ precursors	Resveratrol (Natural), SRT2104 (Synthetic), NAD^+^ precursors (e.g., NMN, NR)	Activates SIRT1, promoting osteoblast differentiation and inhibiting osteoclastogenesis. Boosts NAD^+^ levels to support SIRT1's NAD^+^‐dependent activity. Anti‐inflammatory and antioxidant effects.	Resveratrol in OVX rodents: Improves trabecular bone structure and reduces osteoclast activity via SIRT1/β‐catenin pathway [25]. Resveratrol in human T2DM: Increases SIRT1 in BM‐MSCs, reduces apoptosis, promotes osteogenesis [66]. NAD^+^ precursors: Improve endothelial function and reduce oxidative stress in other tissues, plausibly mitigating vascular leakage [73].	Dose‐dependent effects: High doses of resveratrol may worsen bone loss or adiposity in some models [77]. Bioavailability: Resveratrol has poor bioavailability, requiring advanced delivery systems [78]. Limited direct evidence: Few studies directly test these compounds in BME models.
Combination therapies	SIRT1 activators + Anti‐inflammatory (e.g., IL‐1β, TNF‐α inhibitors) or Anti‐VEGF agents	SIRT1 activation targets metabolic/oxidative stress, while adjunct therapies directly address vascular permeability and inflammatory cytokines for a synergistic effect.	In analogous models (e.g., osteoarthritis), combinations of anti‐inflammatory agents plus resveratrol‐like compounds produce stronger protection than either alone [25]. Theorized to be highly effective for multifactorial BME, though no direct studies exist [74].	Increased complexity: Optimal dosing schedules and potential drug interactions are unknown. Lack of direct evidence: No published studies have tested combination therapies specifically for BME.
Risks and side effects	N/A	Overactivation of SIRT1 or off‐target effects can occur due to systemic action.	Off‐target effects: Chronic high‐dose resveratrol can reduce trabecular bone volume or increase bone resorption markers in some ageing models [77]. Safety profile: Human trials show resveratrol is generally safe with mild adverse events (e.g., GI discomfort), but risks in special populations (e.g., coagulopathies) need assessment [80].	Tissue specificity: Balancing effects in marrow/endothelium against actions in other tissues is challenging. Dose and timing: The effective window for acute versus chronic BME may differ; late intervention could be counterproductive.

**Figure 6:**
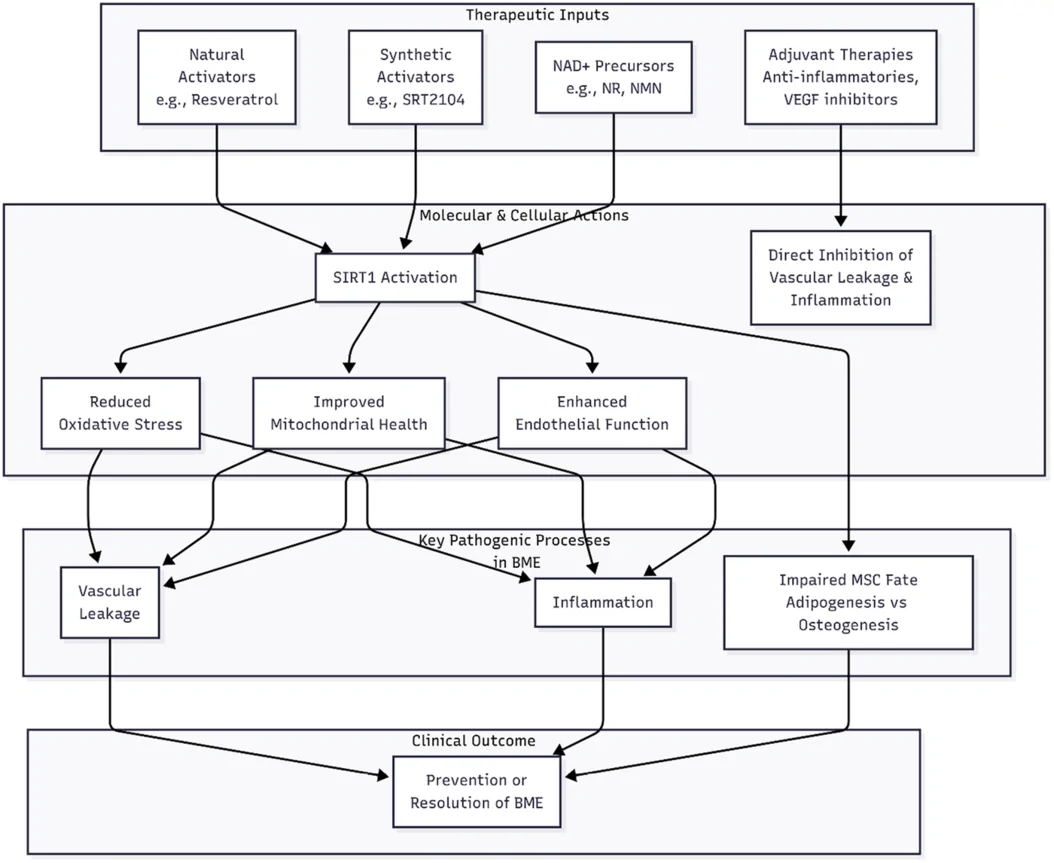
Strategic approach to targeting Bone Marrow Edema (BME) through SIRT1 activation and combination therapy.

Therapeutic modulation of SIRT1 holds promise as a strategy to prevent or treat BME‐like conditions by targeting upstream drivers of edema: inflammation, vascular leakage, oxidative stress, and MSC fate. Resveratrol and related activators already display encouraging effects in preclinical and early clinical studies in bone metabolic disease. However, direct trials in BME are lacking. For translation, key issues include finding suitable dosing and delivery, ensuring tissue targeting, combining with adjunct therapies addressing vascular or inflammatory damage, and monitoring for side effects. Future studies should aim to test SIRT1 activators in animal models of BME, measure imaging or histologic edema parameters, and assess functional outcomes to establish efficacy and safety. Figure 6 illustrates the strategic approach to targeting Bone Marrow Edema (BME) through SIRT1 activation and combination therapy.

## 7 Gaps in Knowledge and Future Directions

### 7.1 Gaps in Knowledge and Future Directions

While SIRT1 is increasingly recognized as a key regulator of bone homeostasis, there is a striking lack of *direct* evidence about its role in bone marrow edema (BME) specifically. No published studies (to date) have measured SIRT1 expression or enzymatic activity in BME lesions in humans (e.g., from biopsies guided by MRI) or in animal models that are established specifically to replicate BME [21]. Most of the existing work addresses broader conditions such as osteoporosis, bone marrow adiposity, or metabolic bone disease, but does not assess edema as a distinct imaging or histologic endpoint.

### 7.2 Imaging + Molecular Correlation in Human Patients

A major gap is human translational work correlating imaging findings of BME (MRI, especially fluid‐sensitive sequences) with molecular markers including SIRT1, its downstream effectors, or surrogate measures (e.g., markers of oxidative stress, vascular permeability, or inflammatory cytokines). For example, studies of MSCs from patients with type 2 diabetes show reduced SIRT1 and impaired osteogenesis (but no direct imaging of edema) [72, 81]. To establish whether SIRT1 is altered in edematous marrow, studies combining MRI or other non‐invasive imaging with marrow biopsies or fluid markers (e.g., in joint disease, osteoarthritis or DRON) would be valuable.

### 7.3 Animal Models of BME + SIRT1 Manipulation

There are animal models showing that caloric restriction or models of energy deficit increase bone marrow adiposity, decrease SIRT1, and degrade bone microarchitecture (e.g., the separation‐based anorexia model (SBA)). In these, resveratrol restores SIRT1 levels and rescues aspects of bone architecture and adiposity [47, 53]. However, these models do not necessarily replicate the fluid accumulation/vascular leakage/inflammation characteristic of BME; often they are more about metabolic and structural bone changes [82, 83, 84, 85]. We lack animal experiments that combine imaging of edema (MRI BME signals) and specific gain or loss of SIRT1 (knockout or overexpression) to see if SIRT1 modulation attenuates or prevents BME lesions.

Importantly, many currently available models examining SIRT1 function in bone rely on metabolic or energy‐deficit paradigms, which do not fully capture the acute vascular and inflammatory features characteristic of many forms of BME (e.g., trauma‐related or osteoarthritic lesions).

### 7.4 Time Dynamics: When Does SIRT1 Modulation Have Effect?

Another unanswered question is *timing*. In the course of diseases associated with BME (e.g. osteoarthritis, trauma, overload, metabolic disease), at what stage is SIRT1 downregulated or becomes limiting? Would early intervention prevent the formation of BME? Or is SIRT1 modulation only effective after edema is established, to accelerate resolution or prevent progression? The SBA model shows changes under chronic energy deficit, but does not clarify whether SIRT1 activation earlier would prevent edema‐like changes or whether there is a critical window. Longitudinal animal studies with repeated imaging (e.g. MRI), to track edema onset, progression, and resolution in relation to SIRT1 expression or activity, are needed.

### 7.5 Interplay With Sclerostin, Adiposity, MSC Metabolism in Establishing Edema

There is evidence that SIRT1 represses Sost (sclerostin), which is a potent inhibitor of bone formation, and that SIRT1 haploinsufficiency increases marrow adipogenesis and reduces bone mass [86]. Also, caloric restriction models show that SIRT1 downregulation is associated with increased adipogenesis (marrow adipose tissue, BMAT), reduced osteogenesis, and poor bone architecture [47, 87].

However, how these components, sclerostin upregulation, bone marrow adipose tissue (BMAT) expansion, and altered mesenchymal stem cell (MSC) metabolism collectively contribute to fluid accumulation or vascular leakage (i.e., edema) remains largely unexplored. Expanded BMAT may influence the marrow microenvironment both mechanically, by occupying limited trabecular space, and biologically, through the secretion of adipokines that could modulate vascular permeability and inflammatory signaling. Similarly, altered MSC metabolism, including shifts toward glycolysis and mitochondrial dysfunction, may impair the capacity of stromal cells to maintain local fluid and metabolic homeostasis.

Within this framework, molecular regulators such as Sost (sclerostin), while well established in controlling osteocyte signaling and bone remodeling, may also have indirect effects on the physical characteristics of bone marrow edema (BME). Suppression of Sost promotes bone formation and alters the balance between bone formation and resorption, leading to changes in trabecular architecture [88] and potentially affecting vascular dynamics within the confined marrow space. Taken together, these structural, metabolic, and cellular alterations may converge to influence intraosseous pressure, a defining feature of BME observed on imaging. Specifically, increased bone turnover, combined with vascular dysregulation and interstitial fluid accumulation, can result in confined expansion within the rigid trabecular compartment, thereby elevating intraosseous pressure. In this context, SIRT1‐regulated pathways may not only shape cellular remodeling but also contribute to the biomechanical and fluid‐dynamic environment underlying BME, although direct evidence linking these processes remains limited and warrants further investigation.

**Figure 7:**
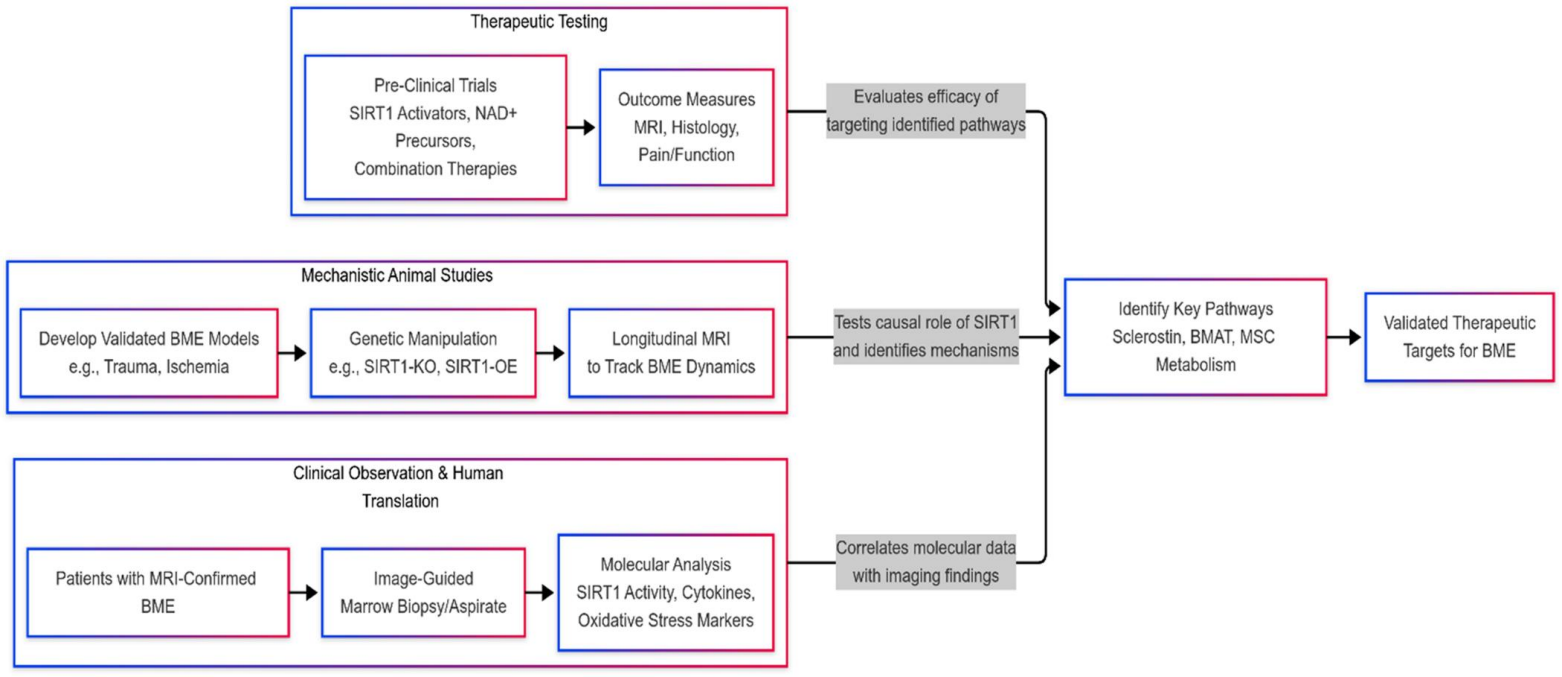
Proposed integrated research strategy to address the critical gaps in understanding SIRT1's role in Bone Marrow Edema (BME). The approach synergizes A) human translational studies to correlate clinical imaging with molecular data, B) mechanistic animal studies using genetically modified models and longitudinal MRI to establish causality and dynamics, and C) pre‐clinical therapeutic testing. The convergence of these streams will help identify the key pathways (e.g., sclerostin, marrow adiposity, MSC metabolism) that link SIRT1 dysfunction to BME, ultimately leading to validated therapeutic targets.

Future research should prioritize bridging the current gap in direct evidence of SIRT1 involvement in BME by combining imaging and molecular biology: in human patients with MRI‐confirmed BME or bone marrow lesions, it is crucial to obtain marrow biopsy or aspirate samples to quantify SIRT1 expression, deacetylase activity, downstream targets, and correlate these with severity, duration and resolution of edema signals. In parallel, animal models that faithfully reproduce BME (e.g., trauma, overload, osteoarthritis, ischemia/venous stasis) should be developed or adapted, coupled with genetic manipulation of Sirt1 (conditional knockout, overexpression or inducible suppression) to assess whether modulating SIRT1 alters onset, progression, or recovery of edema. Time‐course studies are particularly needed to establish when in disease the modulation of SIRT1 is most effective, whether early intervention (pre‐edema or during early fluid accumulation) yields prevention, or whether later activation promotes resolution. Also critical is clarifying the interplay among sclerostin (Sost), marrow adiposity (BMAT), and MSC metabolic shifts as contributors to edema: for example, does upregulation of sclerostin in osteocytes or altered MSC differentiation toward adipocytes promote vascular leakage or intraosseous pressure that triggers edema? And can SIRT1 activation suppress sclerostin or BMAT expansion in ways that measurably reduce fluid accumulation in marrow? Another priority is examining metabolic pathways downstream of SIRT1 in marrow stromal or MSC lineages specifically glycolysis, mitochondrial function, autophagy and redox homeostasis, as these may influence cellular energy demand, ROS generation, ion transport, vascular integrity, and ultimately fluid homeostasis in the marrow space. Finally, translational preclinical and early clinical intervention studies are needed: testing of SIRT1 activators (resveratrol, synthetic agonists), NAD^+^ precursors, or combined therapies (targeting vascular stabilization, inflammation, etc.), using endpoints that include imaging of BME (MRI or advanced CT), histology, pain or function outcomes, and safety/tissue specificity profiles. Addressing these areas of work would substantially clarify whether SIRT1 is not only mechanistically implicated in BME but also a viable therapeutic target in the prevention or treatment of edematous bone marrow lesions. Figure 7 illustrate the proposed integrated research strategy to address the critical gaps in understanding SIRT1's role in BME.

## 8 Conclusion

In summary, substantial evidence supports SIRT1 as an important regulator of bone homeostasis, acting through multiple mechanisms including promotion of osteoblast differentiation and function, suppression of osteoclast activity, regulation of mesenchymal stem cell fate, and maintenance of vascular and metabolic integrity. These well‐established roles position SIRT1 as a key integrator of skeletal remodeling processes.

However, with respect to bone marrow edema (BME), the current body of evidence remains largely indirect. While SIRT1 modulates several biological pathways implicated in BME pathophysiology, such as inflammation, oxidative stress, marrow adiposity, and vascular permeability direct lesion‐level or causal evidence linking SIRT1 dysfunction to the initiation or progression of BME is still limited. Accordingly, rather than being definitively established as a central regulator of BME, SIRT1 should presently be considered a biologically plausible and potentially important mediator within a multifactorial framework of edema development. Its influence is likely context‐dependent and may interact with other pathological drivers, including mechanical stress, vascular compromise, and inflammatory signaling.

Nevertheless, the convergence of mechanistic insights provides a strong rationale for further investigation. Future studies integrating imaging, molecular profiling, and functional models will be essential to determine whether SIRT1 plays a causal role in BME and whether its modulation can offer therapeutic benefit.

In this context, SIRT1 represents a promising but as yet unvalidated target, warranting rigorous experimental and translational evaluation in bone marrow edema and related skeletal disorders. Importantly, the potential for adverse skeletal effects with excessive SIRT1 activation underscores the need for careful therapeutic calibration to avoid unintended promotion of bone resorption.

## Author Contributions

**Yiming Chen:** conceptualization, funding acquisition, writing – original draft, writing – review and editing. **Xinyue Li:** conceptualization, investigation, writing – original draft, visualization, project administration. **Zhongji Zheng:** conceptualization, visualization, writing – review and editing, project administration. **Zhihong Liu:** conceptualization, validation, writing – review and editing, investigation, writing – original draft. **Tao Xu:** investigation, conceptualization, writing – review and editing, writing – original draft, project administration, visualization, resources. **Yuanyi Tang:** conceptualization, validation, writing – review and editing, resources, writing – original draft, funding acquisition, software, data curation, supervision, formal analysis, methodology.

## Generative AI Statement

The author(s) declare that no Generative AI was used in the creation of this manuscript.

## Data Availability

Data sharing not applicable to this article as no datasets were generated or analysed during the current study.
